# Childbirth Preparation: Knowledge of the Use of Non-Pharmacological Pain Relief Methods during Childbirth in Tshwane District, South Africa: A Cross-Sectional Study

**DOI:** 10.3390/nursrep14010001

**Published:** 2023-12-21

**Authors:** Seemole Eniffer Matabane, Maurine Rofhiwa Musie, Mavis Fhumulani Mulaudzi

**Affiliations:** Department of Nursing Science, Faculty of Health Science, University of Pretoria, Gezina 0001, South Africa; maurine.musie@up.ac.za (M.R.M.); mavis.mulaudzi@up.ac.za (M.F.M.)

**Keywords:** childbirth, knowledge, non-pharmacological, pain relief, preparation

## Abstract

Effective management of labour pain is an essential aspect of care provided to women during childbirth. The aim of this study was to assess pregnant women’s knowledge of using non-pharmacological pain relief methods during childbirth. Methods: This quantitative descriptive cross-sectional study was conducted at four hospitals in the Tshwane District of South Africa. The sample consisted of 384 pregnant women. Results: The results show that (*n* = 200, 52.1%) pregnant women lack knowledge regarding non-pharmacological pain relief methods, while a minority (*n* = 101, 26.3%) had some knowledge, some (*n* = 73, 19%) were uncertain about these methods and others (*n* = 10, 2.6%) did not comment. Additionally, most respondents (*n* = 232, 60%) never received education about the various methods available to manage pain during labour at antenatal care, while others (*n* = 131, 34%) agreed that they received such education. A significant relationship between the level of education and knowledge about non-pharmacological pain relief methods was noted (*p* = 0.0082). In conclusion, respondents knew methods such as massage, breathing exercises, movements and birth positions. However, they lacked knowledge of birth companionship, acupuncture, transcutaneous electrical nerve stimulation (TENS), aromatherapy and music. The overall findings revealed that antenatal care services provided by midwives are not effectively preparing pregnant women for pain relief during childbirth.

## 1. Introduction

Labour pain is commonly described as the most intense pain experienced by women during childbirth and presents significant psychological (for example, fear and anxiety) and physiological (e.g., birth positions) challenges [1,2]. Some childbearing women cope well with labour pain without any intervention, whereas others require pharmacological and/or non-pharmacological methods for pain relief [2]. Pharmacological interventions in managing labour pain have been predominant in the field of maternity care owing to their effectiveness in reducing pain [3]. In contrast, non-pharmacological methods are perceived as being less effective [3]. Utilising non-pharmacological pain relief techniques during labour requires pregnant women to begin their preparation during the antenatal period [4]. Non-pharmacological interventions include massage, breathing techniques, positioning, music, hydrotherapy, acupressure and aromatherapy [3]. The World Health Organization (WHO) recommends [5] intrapartum care for a positive childbirth experience and effective pain management as an essential component of the care plan for childbearing women.

Women’s perception of labour pain may be associated with previous childbirth experiences or a fear of childbirth (FOC) [6]. In addition, for some women, childbirth can pose a threat and cause harm and pain that can generate intense fear, mostly in nulliparous women. The experience of fear usually generates a maternal psychological discomfort, which causes an increase in catecholamines, preventing the normal secretion of oxytocin that is meant to trigger the correct initiation of labour, thus leading to prolonged labour [1,7]. Midwives are responsible for preparing women for labour pain during information sessions at antenatal visits and providing information on various options, including pharmacological and non-pharmacological pain relief methods [1]. In contrast, ref. [7] identified different potential causes, such as inadequate awareness, knowledge deficit, lack of social support and other women’s negative stories, contributing to a lack of knowledge on various non-pharmacological methods available. The presence of FOC has several risks, including increased chances of abortion, post-traumatic stress disorder, depression and the risk of birth complications like fistula, dystocia, hypertension and preeclampsia. Hence, effective pain management has become an essential aspect of the care provided to women during childbirth [1,6,7].

Globally, pharmacological interventions are frequently used during labour and childbirth [7]. The commonly used pharmacological method is opioids, particularly pethidine [7]. This method helps women to relax and cope with pain due to intense uterine contractions. However, this method is associated with adverse side effects, which include nausea, sedation and a negative impact on women’s ability to breastfeed [8,9] safely. Ref. [10] reports that most women experience mistreatment during childbirth. That is, they encounter caregivers who do not incorporate emotional needs into their care and withdraw pain relief during labour, which is seen as mistreatment or, in some instances, as a form of abuse or obstetric violence [10]. The current approaches to alleviate labour pain encompass pharmacological and non-pharmacological interventions [5,10]. Failure to adequately manage labour pain can have negative consequences on the well-being of mothers, potentially impacting the baby and the family [11].

Similarly, the authors of [12] indicated that FOC and its complications would likely increase obstetric interventions and subsequent healthcare costs from 5% to more than 40%. A study conducted in Australia indicated that non-pharmacological methods of pain relief during labour can enhance childbirth satisfaction by providing cognitive, physical and psychological support throughout the delivery process. As a result, various childbirth education approaches have included non-pharmacological techniques [13]. Non-pharmacological options include emotional support, directed breathing and relaxation techniques, massage, labouring in water and transcutaneous electrical nerve stimulation (TENS) [5,14]. Additionally, the authors of [4] indicated that non-pharmacological interventions include cognitive behavioural therapy, relaxation therapy, biofeedback, patient education, self-management and social support interventions. The effective use of these non-pharmacological methods is associated with knowledge and guidance [15]. Still, the study revealed that most pregnant women (83.3%) did not have precise information regarding the techniques used during the different stages of labour and childbirth [15].

A study conducted in Brazil highlighted that there is a desire among women to receive guidance on non-pharmacological pain relief methods for labour control [16]. In Africa, the utilisation of non-pharmacological pain management during vaginal childbirth is notably low, with only 0.3% of women opting for such methods [7]. Over 86% of pregnant women in Nepal wanted their pain to be relieved during childbirth [17]. However, the scarcity of non-pharmacological pain relief utilisation was reported globally. Additionally, ref. [13] indicates that, in South Africa, midwives are responsible for providing maternity care. Still, they have kept on violating the rights of pregnant women by denying them pain relief during childbirth. Hence, the objective of the study was to assess pregnant women’s knowledge of the non-pharmacological pain relief interventions utilised during childbirth using a quantitative approach. This study was conducted to support guidance on pain relief as part of an evidence-based preparation for the WHO’s recommendations on intrapartum care for a positive childbirth experience [5].

## 2. Materials and Methods

### 2.1. Study Design

This research study employed a cross-sectional descriptive quantitative design involving numerical presentation and data analysis using statistical rules [18,19]. The cross-sectional design involved human participants facing challenges [20].

### 2.2. Participants

The sample for this study comprised 384 pregnant women in their reproductive years. Stratified random sampling was used, with the selected district hospitals categorised into different regions within the Tshwane municipality. Stratified random sampling was employed to ensure respondents from each selected district hospital were adequately represented. The size of the strata was determined in proportion to the hospital population size within each stratum. The below formula was employed:

$$n_{i}=n.\frac{\;N_{i}}{N}$$

*N* refers to the population size.

*N_i_* _=_ population size of each stratum.

*n_i_* = required sample size for each stratum.

*n* = sample size.

The admission register randomly selected patients according to the inclusion criteria.

Samples from each stratum were added to form a complete sample size of 384. Hospital 1 in region 1 (*n* = 81), hospital 2 in region 6 (*n* = 159), hospital 3 in region 2 (*n* = 73) and hospital 5 in region 3 (*n* = 71) represented the respective strata. The respondents were recruited during antenatal care (ANC) follow-up visits. Pregnant women with a previous obstetric history of Caesarean section delivery and those who delivered normally but had a history of stillbirth or early neonatal death were excluded from the study. Pregnant women with a history of previous Caesarean section were excluded because the researcher assumed some might not have experienced labour pain.

### 2.3. Instrument

Participants voluntarily completed a self-administered questionnaire comprising both open-ended and closed-ended questions. Closed-ended questions included response options of “yes”, “no”, and “not sure”, while open-ended questions required written responses. A five-point Likert scale was used to assess knowledge of different non-pharmacological methods, with respondents selecting only one answer. The Likert scale questions also measured varying levels of agreement and disagreement. The questionnaire was developed based on the existing literature and piloted in one of the selected district hospitals to ensure validity and reliability [11,14,15,18]. The questionnaire was divided into sections, including demographic information (5 items), knowledge of non-pharmacological pain relief methods (11 items), types of non-pharmacological methods (12 items), effectiveness of ANC (4 items) and suggestions for pain relief methods which consisted of (3 items). The total scale score was achieved by adding up the values assigned to each item; the higher the score, the higher the level of knowledge.

A pilot study was conducted in one of the four selected district hospitals. According to [18], the aim of a pilot study is to assist with identifying problems that may interfere with study validity. Only 5% of the sample size was selected to pre-test the tool before a complete study. Errors were placed in the data collection tool, and the tool was revised. The piloting results were not included in the results of the main study.

### 2.4. Procedure

The researcher ensured that respondents gave consent before answering the questions. To recruit the respondents, the researcher visited the ANC clinics of selected hospitals early in the morning and utilised the platform where the midwives were addressing and giving health education to the mothers. Pregnant women at the Antenatal clinic were informed about the study’s aims and objectives. Ensuring effective recruitment is essential to avoid ethical consequences and financial implications [21]. Only those who showed interest met the inclusion criteria, and those who were willing to sign the consent form were given the questionnaire. The questionnaire was distributed from 1 September to 7 November 2022, and the researcher stayed with them until they completed it without influencing their opinion. The STROBE guidelines were used to report the study (Supplementary File S1).

### 2.5. Data Analysis

The data were manually captured in an Excel spreadsheet and sent to the statistician for cleaning and analysis using the Statistical Package for the Social Sciences (SPSS (https://www.ibm.com/products/spss-statistics)). Descriptive statistics, such as frequencies and percentages, were utilised to present the data, which were then visualised in tables and graphs [18,21]. Graphs and tables were used to present the numerical data, and a statistician conducted the data analysis using statistical principles. Additionally, the Chi-Square test was employed to determine relationships between variables. The association between variables was examined by interpreting the *p*-value, which indicates the significance of the relationship [18].

### 2.6. Ethical Considerations

This study received ethical approval from the University Faculty of Health Care Science Research Ethics Committee, with reference number 236/2022. Additionally, permission was obtained from the management of the district hospital and the institution’s research committee. The ethical principles that guided this study included the Nuremberg Code, the Declaration of Helsinki, and the South African National Health Research Ethics Council. Before their participation, the pregnant women were invited to participate in the study after providing informed consent. They were explicitly informed that their participation was voluntary and that they had the right to withdraw from the study at any point. This study adhered to justice, respect, beneficence, anonymity and confidentiality.

## 3. Results

The mean age of respondents was (30.55, SD = 6.205). The majority of respondents (*n* = 102, 26.6%) were between the age of 31 and 35 years, followed by (*n* = 99, 25.8%) of respondents falling under the age group of more than 35 years, while (*n* = 95, 24.7%) were between the ages of 26 and 30 years and (*n* = 88, 22.9%) were less than 25 years. The sample size per institution was as follows: hospital 1 (*n* = 81), hospital 2 (*n* = 159), hospital 3 (*n* = 73) and hospital 5 (*n* = 71).

In terms of parity majority of respondents, 30.5% (*n* = 117) had two children, followed by women with one child, 26.3% (*n* = 101) and Primigravida 21.6% (*n* = 83), Para 3, 15.9% (*n* = 61), Para 4, 4.2% (*n* = 16), Para 5, 1.3% (*n* = 5), and Para 6, 0.3% (*n* = 1). African language was the most dominant language at 89.3% (*n* = 343), followed by English 4.4% (*n* = 17) and Afrikaans 1.3% (*n* = 5). Furthermore, 4.9% (*n* = 19) did not comment.

Pregnant women who delivered normally were 65.9% (*n* = 253). Those who were pregnant for the first time were 21.6% (*n* = 83) and 6.8% (*n* = 26) were pregnant women who delivered vaginally after Caesarean section. A total of 5.7% (*n* = 22) did not comment. The sociodemographic data can be consulted in Table 1.

### 3.1. Pregnant Women’s Knowledge Regarding Non-Pharmacological Pain Relief

The results indicate that (*n* = 200, 52.1%) of pregnant women had never heard about non-pharmacological pain relief methods. Meanwhile, (*n* = 101, 26.3%) had heard about it, while about (*n* = 73, 19. %) were not sure about whether they had ever heard about it or not. About (*n* = 10, 2.6%) did not comment.

### 3.2. Option to Use Non-Pharmacological Pain Relief

Some pregnant women (*n* = 128, 33.3%) were willing to opt for non-pharmacological pain relief during labour. Meanwhile, (*n* = 99, 25.8%) were sure that they would not opt for these methods, while a minority (*n* = 31, 8.1%) decided not to comment and (*n* = 126, 32.8%) were not sure. See Table 2.

### 3.3. Benefits of Non-Pharmacological Pain Relief

Some respondents (*n* = 132, 34.0%) expressed a willingness to recommend non-pharmacological pain relief methods to their friends. However, majority at (*n* = 181, 47%), reported being unaware of the benefits associated with these methods. Similarly, (*n* = 181, 47%) indicated a lack of knowledge regarding the disadvantages of non-pharmacological pain relief methods. Some respondents also mentioned concerns that these methods may not effectively relief pain and could have the potential of harming the baby. See Table 3.

### 3.4. Types of non-Pharmacological Methods Used during Labour

The findings revealed that the majority of respondents (*n* = 133, 35.0%) reported having acquired knowledge about massage as a method of pain relief. Similarly, (*n* = 118, 31%) agreed they had learned about hydrotherapy. Regarding homoeopathy, the majority of respondents (*n* = 203, 52%) were uncertain about their knowledge. On the other hand, (*n* = 110, 28%) disagreed with having acquired information about aromatherapy, while (*n* = 105, 27%) disagreed with music therapy being a pain relief method. In contrast, a majority of respondents (*n* = 194, 50%) agreed that they had learned about breathing exercises for pain relief. Regarding acupuncture, (*n* = 207, 54%) of respondents were uncertain. Regarding the superficial application of heat and cold on the lower abdomen, most respondents (*n* = 191, 48%) were uncertain, while (*n* = 78, 21%) disagreed that they learnt about this method. Similarly, (*n* = 177, 46%) of respondents were unsure about transcutaneous electrical nerve stimulation (TENS), with (*n* = 118, 31%) disagreeing and (*n* = 60, 16%) strongly disagreeing with having knowledge about it. On the other hand, (*n* = 163, 42%) of respondents agreed that they had learned about movement and birth position changes as methods of pain relief. Table 4 shows that the majority of the respondents (*n* = 232, 60%) indicated that they were never educated on the types of methods, which supports the reason for the uncertainty of respondents in terms of the methods indicated above.

### 3.5. Preparation of Labour

The Table 5 below illustrate that 17.1% (*n* = 66) of the pregnant women preferred that labour preparation be conducted in the form of teaching moment. This is followed by those who indicated that they do not have any idea at 9.4% (*n* = 36). The majority at 67.4% (*n* = 259) did not comment.

According to the results, it is evident that pregnant women expect to be given more information in teaching during antenatal visits.

Table 6 illustrates the association between types of non-pharmacological pain relief methods and education. The results reveal that acupuncture, aromatherapy, hydrotherapy and superficial application of heat and cold were significant when associated with education. This implies that these methods are utilised depending on respondent’s level of education. All other methods, such as TENS, homoeopathy, massage, music, breathing exercises, movement and doula, were not significantly associated with education. This implies that during the clinical session, women should be given more information on non-pharmacological pain relief methods.

A significant relationship between level of education and knowledge of non-pharmacological pain relief methods was noted in this study (*p* = 0.0082). The results reveal that most respondents across all levels of education had never heard about these methods. Pregnant mothers cannot utilise these methods because they are not known. See Table 7.

## 4. Discussion

The study assessed pregnant women’s knowledge regarding non-pharmacological pain relief methods utilised during labour. The demographic data of the respondents who participated in the study were age, level of education and women’s parity. Most respondents were between the ages of 31 and 35, with matric as the highest level of education. However, a study conducted in Brazil revealed different demographic data of respondents between the ages of 15 and 35, with post-matric as the highest level of education [22]. The findings indicate that pregnant women lack information regarding some of the available non-pharmacological methods during labour and childbirth. The results align with a study conducted in India, where only a minimal percentage of pregnant women, at 10%, were aware of non-pharmacological pain relief [23]. A lack of knowledge influences their decision to make informed choices during labour. The results indicate that (*n* = 200, 52.1%) of pregnant women have never heard about non-pharmacological pain relief methods. Non-pharmacological pain relief methods for pregnant women are often used as alternatives or complementary approaches to manage pain during labour and childbirth. This finding suggests a lack of awareness or education among pregnant women regarding alternative pain management techniques during labour and childbirth. According to [8,18], non-pharmacological pain relief methods were perceived as time-consuming and did not relieve pain, and women in Europe believed that these methods were not concrete. Furthermore, ref. [24] indicated that the barrier to the utilisation of non-pharmacological pain relief methods is a lack of knowledge by pregnant women and a lack of interest by health care providers.

Pregnant women (*n* = 128, 33.3%%) are willing to opt for non-pharmacological pain relief. Again, it was found that some respondents (*n* = 132, 34%) were willing to recommend non-pharmacological pain relief methods to their friends. The willingness of these women to consider non-pharmacological pain relief methods indicates the potential benefits and advantages of these methods to be utilised. This finding aligns with WHO intrapartum care recommendations, emphasising non-pharmacological pain relief methods. However, a recent European study revealed that women are not willing to utilise pain relief during labour and childbirth [16]. However, the majority at (*n* = 181, 47%%) reported being unaware of the benefits associated with these methods.

Similarly, (*n* = 181, 47%) of respondents indicated a lack of knowledge regarding the disadvantages of non-pharmacological methods. A lack of knowledge was identified as the primary obstacle preventing the utilisation of non-pharmacological pain relief methods [16]. Patient education was suggested to be the key method of health promotion that can be used to address the lack of knowledge and promote awareness about pain management [10,25].

Most respondents (*n* = 232, 60%) indicated that they were never educated on types of methods. Similarly, ref. [8] reported that there is scarce information and that there are gaps regarding the knowledge on what pregnant women know about non-pharmacological techniques for pain relief. Addressing the gap in knowledge and awareness about non-pharmacological pain relief methods among pregnant women is crucial. Healthcare professionals and childbirth educators can be vital in providing accurate information, promoting education and raising awareness about these techniques. This can empower pregnant women to make informed decisions and actively participate in their birth experience. To support the above information, ref. [1] suggested that theoretical and practical non-pharmacological pain management interventions should be incorporated in nursing curricula and advocated for the importance of non-pharmacological pain management methods.

Additionally, refs. [16,24] highlight that the role and benefits of non-pharmacological pain relief are crucial and cannot be ignored. According to [22,24,26], non-pharmacological pain relief methods benefit both the mother and the neonate.

By offering pain management options and ensuring that women have access to comprehensive information, healthcare providers can support women in making choices that align with their preferences and needs. This may lead to improved birth experiences, increased satisfaction, reduced midwifery litigation and reduced reliance on pharmacological interventions [2,7,10].

### Limitations

This study was about the knowledge of non-pharmacological pain relief methods available during labour conducted in Tshwane district hospitals. However, this study was limited to only four district hospitals and pregnant women attending clinics at provincial district hospitals. The findings excluded pregnant women who attended antenatal care in private institutions. Therefore, the study recommendations will only apply to public institutions’ maternity areas.

## 5. Conclusions

In conclusion, a certain percentage of pregnant women are unaware of non-pharmacological pain relief methods. However, some pregnant women are willing to utilise these methods. The respondents also highlighted a need for teaching sessions during antenatal visits. Efforts should be made to bridge the knowledge gap and promote education, enabling women to make informed decisions and access a broader range of pain management techniques during labour and childbirth.

Healthcare providers, specifically midwives, should organise educational sessions aimed at improving the understanding of non-pharmacological pain relief methods among pregnant women. These sessions should offer a comprehensive overview of these methods’ benefits, barriers, advantages and disadvantages. Despite being endorsed by the World Health Organization (WHO), many healthcare facilities do not routinely provide these methods due to potential barriers and the necessity for midwives to undergo appropriate training.

## Figures and Tables

**Table 1:** Sociodemographic data.

Sociodemographic Data		
**Age**	**Frequency (*n*)**	**Percent (%)**
<25 years	88	22.9
26–30 years	95	24.7
31–35 years	102	26.6
>35 years	99	25.8
**Parity**	**Frequency (*n*)**	**Percent (%)**
0	83	21.6
1 normal vertex delivery	101	26.3
2 normal vertex delivery	117	30.5
3 normal vertex delivery	61	15.9
4 normal vertex delivery	16	4.2
5 normal vertex delivery	5	1.3
6 normal vertex delivery	1	0.3
**Home language**	**Frequency (*n*)**	**Percent (%)**
**African**	**343**	**89.3**
Afrikaans	5	1.3
English	17	4.4
No comment	19	4.9
**Level of education**	**Frequency (*n*)**	**Percent (%)**
No schooling (illiterate)	15	3.9
Some schooling (did not complete matric)	74	19.3
Matric	173	45.1
Post-matric	101	26.3
No comment	21	5.5
**Total**	**384**	**100%**

**Table 2:** Option to use non-pharmacological pain relief.

Will You Opt for Non-Pharmacological Pain Relief during Labour?	Frequency (*n*)	Percent (%)
No	99	25.8
No comment	31	8.1
Not sure	126	32.8
Yes	128	33.3
**Total**	**384**	**100%**

**Table 3:** Benefits of non-pharmacological pain relief methods.

What Are the Benefits of Non-Pharmacological Pain Relief Methods?	Frequency (*n*)	Percent (%)
I don’t know	181	47
No benefits	3	1
No comment	31	8
Not sure	93	24
It’s a natural method	5	1
Makes birth easy	9	2
No side effects	11	3
Relieve pain	51	13
**Total**	**384**	**100%**

**Table 4:** Information on types of non-pharmacological pain relief methods.

Did the Midwife (Registered Nurse) Educate You on Types of Methods That Can Be Used to Control Pain during Labour?	Frequency (*n*)	Percent (%)
No comment	21	6.0
No	232	60.0
Yes	131	34.0
**Total**	**384**	**100%**

**Table 5:** Labour preparation.

How Do You Think Labour Preparation Can Be Conducted	Frequency (*n*)	Percent (%)
Have no idea	26	6.8
Have teaching moment	66	17.1
I think is fine the way it is	1	0.3
No comment	259	67.4
Not sure	23	6.0
By being patient	2	0.5
Do exercise during follow ups	5	1.3
Forming groups during visit	1	0.3
Have private place for consultation	1	0.3
**Total**	**384**	**100**

**Table 6:** Association between non-pharmacological pain relief methods and education.

Type of Methods	Level of Education	Strongly Agree	No Comment	Agree	Not Sure	Disagree	Strongly Disagree	*p*-Value *
Massage	Matric	8	4	37	30	12	9	None
	No comment	10	0	33	38	9	10	
	No schooling	13	7	40	33	7	0	
	Post-matric	11	2	39	27	12	9	
	Some schooling	3	1	21	49	11	15	
Hydrotherapy	No comment	0	5	5	52	29	9	*p* = 0.0212 *
	No schooling	13	7	40	33	7	0	
	Some schooling	3	2	21	49	11	14	
	Matric	8	4	37	30	12	9	
	Post-matric	11	2	39	27	12	9	
Homeopathy	No comment	0	4	10	38	38	10	None
	No schooling	0	7	7	60	13	13	
	Some schooling	1	0	7	60	20	12	
	Matric	2	1	11	55	24	7	
	Post-matric	2	0	7	46	34	12	
Aromatherapy	No comment	0	10	0	52	28	10	*p* = 0.0071
	No schooling	0	20	7	47	13	13	
	Some schooling	0	3	11	48	30	8	
	Matric	2	2	12	45	29	10	
	Post-matric	3	1	22	33	29	12	
Music therapy	No comment	5	9	19	29	33	5	None
	No schooling	7	20	7	40	26	0	
	Some schooling	2	11	20	31	28	8	
	Matric	8	4	25	28	24	11	
	Post-matric	9	4	20	24	31	12	
Breathing exercise	No comment	10	10	33	33	4	10	None
	No schooling	7	7	40	20	13	13	
	Some schooling	10	3	48	23	10	7	
	Matric	16	2	54	12	6	10	
	Post-matric	24	2	50	9	6	9	
Acupuncture	No comment	0	0	24	62	9	5	*p* = 0.0088 *
	No schooling	20	7	13	40	20	0	
	Some schooling	0	7	8	54	20	11	
	Matric	5	4	9	57	19	7	
	Post-matric	2	0	10	50	30	9	
Superficial application of heat and cold	No comment	0	0	14	58	14	14	*p* = 0.0350 *
	No schooling	7	7	0	60	7	19	
	Some schooling	1	0	11	61	19	8	
	Matric	4	1	20	51	17	7	
	Post-matric	6	1	14	36	30	13	
TENS	No comment	0	5	5	48	33	9	None
	No schooling	0	0	7	73	0	20	
	Some schooling	0	3	5	58	23	11	
	Matric	1	1	8	45	30	15	
	Post-matric	1	1	2	35	40	21	
Movement and position changes	No comment	5	2	45	29	11	8	None
	No schooling	5	0	57	33	5	0	
	Some schooling	7	0	40	20	13	20	
	Matric	6	1	41	31	16	5	
	Post-matric	8	1	34	34	18	5	
Birth ball	No comment	5	13	10	57	10	5	None
	No schooling	12	0	27	47	7	7	
	Some schooling	3	7	13	53	15	9	
	Matric	3	4	24	42	20	7	
	Post-matric	7	1	28	39	19	6	
Birth companion	No comment	14	0	24	48	14	0	None
	No schooling	13	7	26	47	7	0	
	Some schooling	1	3	15	54	18	9	
	Matric	8	2	17	47	19	7	
	Post-matric	9	1	16	40	26	8	

* Probability value determined by the significance test.

**Table 7:** Association between education and having heard of non-pharmacological pain relief methods knowledge.

Education	No	No Comment	Not Sure	Yes	Total (%)	*p*-Value
No comment	38	5	33	24	100	
No schooling	40	20	13	27	100	***p* = 0.0082**
Some schooling	57	4	18	21	100	
Matric	50	1	20	29	100	
Post-matric	56	1	17	26	100	

## Data Availability

The data presented in this study are available on request from the corresponding author.

## Supplementary Materials

The following supporting information can be downloaded at: https://www.mdpi.com/article/10.3390/nursrep14010001/s1, File S1: STROBE checklist of items included in the study.

## Public Involvement Statement

There was no public involvement in this research.

## Guidelines and Standards Statement

This manuscript was drafted utilizing the Strengthening the Reporting of Observational studies in Epidemiology (STROBE) guidelines for cross-sectional studies. These guidelines can be accessed via the Equator Network: https://www.equator-network.org (accessed on 19 October 2023).
