# Supplementary material for: Childbirth Preparation: Knowledge of the Use of Non-Pharmacological Pain Relief Methods during Childbirth in Tshwane District, South Africa: A Cross-Sectional Study
Source: Nurs Rep. 2023 Dec 21;14(1):1–11. doi: 10.3390/nursrep14010001 (PMC10974809; doi:10.3390/nursrep14010001)
Supplement: Supplementary file 1 [file nursrep-14-00001-s001.zip › nursrep-2668007-supplementary.pdf]

STROBE checklist of items included in the study

	Item No	Recommendation
**Title and abstract**	1	(*a*) Study design is indicated in the title
		(*b*) Abstract provided summary of how the study was conducted and summary of the results
**Introduction**		
Background/rationale	2	The researcher noted poor utilisation of non-pharmacological pain relief methods for pregnant women during labour. The study aimed to assess pregnant women's knowledge on available non-pharmacological pain relief methods. These methods for pain relief, which do not involve medication, are not being optimally utilized and these methods were recommended globally by the World Health Organisation.
Objectives	3	Objective of the study was to assess pregnant women knowledge on the use of non-pharmacological pain relief methods during childbirth
**Methods**		
Study design	4	The study is quantitative descriptive cross- sectional study
Setting	5	Study design was stated that the study was conducted in district hospital Tshwane South Africa
Participants	6	Participants were pregnant women attending antenatal care in public district hospitals. Stratified random sampling was employed.
Study size	7	The study participants were 384 pregnant women
Statistical methods	8	The statistical method employed were descriptive methods
**Results**		
Main results	9	The main results showed that pregnant women lack knowledge regarding available non-pharmacological pain relief methods available during labour
**Discussion**		
Key results	10	Key results were summarised in the abstract
Limitations	11	The study was limited to pregnant women attending antenatal care in district public hospitals
Interpretation	12	Graphs and tables were used to present findings of the study
Generalisability	13	The study result could not be generalised to all district public hospitals because the study was only conducted in four district hospitals
**Other information**		
Funding	14	The study received no funding,
